# Research Progress on Natural Plant Molecules in Regulating the Blood–Brain Barrier in Alzheimer’s Disease

**DOI:** 10.3390/molecules28227631

**Published:** 2023-11-16

**Authors:** Weidong Wu, Jiahao Huang, Pengfei Han, Jian Zhang, Yuxin Wang, Fangfang Jin, Yanyan Zhou

**Affiliations:** 1 Basic Theory of Chinese Medicine, Heilongjiang University of Chinese Medicine, Harbin 150040, China; wuwd0116@163.com (W.W.); 18845055805@163.com (J.Z.); 18845160421@163.com (Y.W.); 2 Department of Chinese Pharmacology, Heilongjiang University of Chinese Medicine, Harbin 150040, China; hjh123886@163.com; 3 Science and Education Section, Zhangjiakou First Hospital, Zhangjiakou 075041, China; han2444846394@163.com; 4 Department of Internal Medicine, Heilongjiang University of Chinese Medicine, Harbin 150040, China

**Keywords:** natural plant molecules, Alzheimer’s disease, blood–brain barrier, mechanisms, treatment, research progress

## Abstract

Alzheimer’s disease (AD) is a prevalent neurodegenerative disorder. With the aging population and the continuous development of risk factors associated with AD, it will impose a significant burden on individuals, families, and society. Currently, commonly used therapeutic drugs such as Cholinesterase inhibitors, N-methyl-D-aspartate antagonists, and multiple AD pathology removal drugs have been shown to have beneficial effects on certain pathological conditions of AD. However, their clinical efficacy is minimal and they are associated with certain adverse reactions. Furthermore, the underlying pathological mechanism of AD remains unclear, posing a challenge for drug development. In contrast, natural plant molecules, widely available, offer multiple targeting pathways and demonstrate inherent advantages in modifying the typical pathologic features of AD by influencing the blood–brain barrier (BBB). We provide a comprehensive review of recent in vivo and in vitro studies on natural plant molecules that impact the BBB in the treatment of AD. Additionally, we analyze their specific mechanisms to offer novel insights for the development of safe and effective targeted drugs as well as guidance for experimental research and the clinical application of drugs for the prevention and treatment of AD.

## 1. Introduction

Alzheimer’s disease (AD) is a prevalent neurodegenerative disorder characterized by progressive and insidious development, leading to behavioral and cognitive dysfunction. The aging global population and the associated risk factors have contributed to an alarming increase in the number of individuals affected by AD, with projections estimating over 150 million cases worldwide by 2050. This escalating prevalence poses a significant global health challenge, impacting both individuals and society [1,2,3]. Consequently, there is a pressing need to identify safe and effective preventive and control measures.

The pathogenesis of AD remains incompletely understood, primarily due to the intricate interplay between genetic and environmental factors, as well as the diverse biological pathways involved in disease onset and progression. However, certain neuropathological features of AD, such as amyloid plaques and neurofibrillary tangles (NFTs), have been consistently observed. Moreover, emerging evidence suggests that disruption of the blood–brain barrier (BBB) plays a crucial role in AD. BBB breakdown allows neurotoxic blood-derived products, cells, and pathogens to infiltrate the brain, triggering inflammatory and immune responses that impair neuronal and microvascular function. These processes can initiate neurodegenerative pathologies through various pathways. The BBB also restricts the entry of drugs into the brain, posing challenges for targeted drug delivery to the brain. This limitation hinders the development of therapeutic drugs [4,5]. Currently, the US FDA has only approved cholinesterase inhibitors and *N*-methyl-d-aspartate antagonists for the treatment of AD, despite their high incidence of adverse drug reactions. These drugs also have limitations in terms of cost and tolerance. Meanwhile, other drugs with targeted mechanisms are either undergoing clinical trials or have failed to enter the market [6,7,8]. So far, many therapeutic strategies in modern medicine have been unsuccessful in intervening in the disease process and have only been able to manage symptoms [4]. Therefore, the search for a safe and effective drug-targeting pathway and the development of multifunctional and multitargeted drugs for the progression of AD within the complex biological pathways is both challenging and promising.

In recent years, as research on the mechanism of natural plant molecular therapy for AD has advanced, its unique therapeutic advantages have become increasingly apparent. For instance, these molecules can specifically target the brain and possess characteristics such as being multi-targeting, multi-channeling, and having minimal side effects. Specifically, certain monomers and components of traditional Chinese medicine can be combined to offer personalized and precise treatment strategies. However, the specific role of natural plant molecules in regulating the BBB, with their complex pathways and mechanisms of action, has not been clearly explained. This paper aims to comprehensively explore the role of natural plant molecules in regulating the BBB of AD. It will achieve this by systematically reviewing the structure and function of the BBB, the relationship between the BBB and the pathological aspects of AD, the specific mechanisms through which the BBB influences the progression of AD, and the pathways by which natural plant molecules regulate the BBB in AD. The findings will contribute to the identification of a new therapeutic target for the prevention and treatment of AD, and will also serve as a valuable reference for future experimental research and clinical applications.

## 2. Structure and Function of the Blood–Brain Barrier

**Figure 1:**
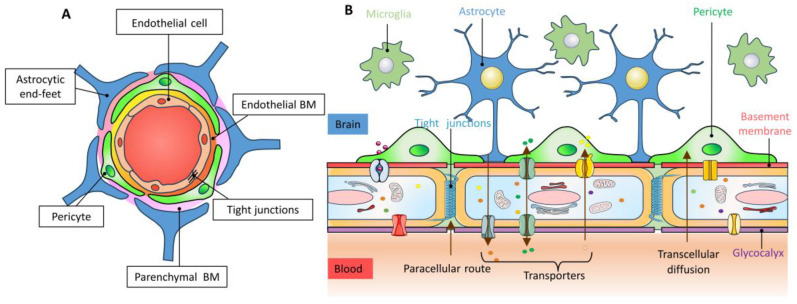
The physiological structure of the blood–brain barrier: (**A**) Cross-sectional structure of the blood–brain barrier; (**B**) Continuity plane structure of blood–brain barrier.

The BBB serves as a dynamic interface between the peripheral circulation and the central nervous system (CNS). It primarily consists of brain capillary endothelial cells, which are surrounded by the basement membrane (BM), pericytes, and astrocytes in the neurovascular unit (NVU). Some studies also consider the NVU as a fundamental component of the BBB (Figure 1) [9,10]. The BBB plays a crucial role in regulating molecular exchanges between blood flow and the brain parenchyma, thereby maintaining CNS homeostasis [11].

### 2.1. Endothelial Cell

Brain microvascular endothelial cells (BMECs) are responsible for maintaining the structural and functional integrity of the BBB. This is achieved through the presence of tight junctions (TJs) and adherens junctions (Figure 1A), which create distinct luminal (apical) and basolateral compartments [12]. The TJ complexes between CNS endothelial cells form a paracellular diffusion barrier for small hydrophilic molecules and ions [13,14,15]. Additionally, the high transendothelial resistance limits the passage of transcellular vesicles through the vessel wall [16]. The influx and efflux of specific substrate macromolecules and proteins within the brain are regulated by passive and active receptors/channels in the lumen or on the lumen surface (Figure 1B) [17,18,19]. As a communication interface between the CNS and the peripheral nervous system, BMECs play a crucial role in regulating circulation, immunity, and controlling the entry of cells and other substances into the brain microenvironment [20].

### 2.2. Astrocytes

Astrocytes, a type of astrocytic glial cells distributed throughout the CNS, play a crucial role in maintaining brain homeostasis and neurological function [21]. They have the ability to chemically and physically interact with neuronal synapses and cerebral microvasculature (Figure 1B). There are several key functions of astrocytes. Firstly, they maintain and regulate the BBB, which is achieved by upregulating the expression of transporter proteins, polarized localization, and specialized enzyme systems, thus promoting TJ stability [22]. Secondly, astrocytes act as critical regulators of the immune response in the CNS. They are involved in phagocytosis, antigen presentation, and the transportation of immune cells [23,24,25]. These functions can either promote or inhibit neuronal damage and inflammation. Additionally, astrocytes interact with microglia to coordinate defense responses [26]. Thirdly, astrocytes can protect neurons by secreting brain-derived neurotrophic factor (BDNF), glial-cell-derived neurotrophic factor (GDNF), and various antioxidants [27]. Fourthly, astrocytes are also involved in neurotransmission. They regulate astrocyte excitability through the activation of cell surface receptors and transporter proteins (Figure 1B). This leads to dynamic changes in intracellular calcium (Ca^2+^) or sodium (Na^+^), as well as changes in ionic and electrical properties of astrocytes. These changes have significant non-autonomous effects on brain function, including the regulation of synaptic activity, neuronal metabolism, and regional blood supply [28]. Finally, astrocyte-derived, wingless-related integration sites (Wnts) play a crucial role in maintaining the activity of Wnt/β-catenin in both endothelial cells and astrocytes. This, in turn, controls the expression of Cav-1, regulates vesicle abundance, and ensures the integrity of terminal feet in the NVU. These processes are essential for maintaining the proper functioning of the endothelial BBB on neurons [29]. Additionally, astrocytes near cerebral microvessels are abundant in aquaporin-4 (AQP4), which is responsible for the uptake and degradation of amyloid β-protein (Aβ) [30,31].

### 2.3. Pericyte

Pericytes are parietal cells that line the septum of the capillary wall [32]. They are recruited by endothelial cells in new microvessels through signal transduction between platelet-derived growth factor-BB (PDGF-BB) and its pericyte receptor PDGFRβ [33,34]. Pericytes are embedded in the BM and attach to the surface of endothelial cells (Figure 1A), including capillaries, small precapillary arterioles, and small postcapillary veins [35,36]. Their high coverage in neural tissue determines their central role in the NVU (Figure 1) [37,38,39,40,41]. Pericytes interact with various components of the NVU, promoting the development of the BBB by supporting the germination, differentiation, and maturation of endothelial cells. This interaction induces the formation of TJs and pericytes also contribute to the construction of part of the BM (Figure 1B). In summary, pericytes play a crucial role in the development and maintenance of the BBB. Under inflammatory conditions, pericytes stimulate immune cells to produce cytokines and present antigens, which inhibits the infiltration of immune system cells into the CNS. Pericytes also guide the astrocyte end foot process towards the endothelial tube, initiate its polarization, and support proper neuronal function [42,43,44]. Additionally, pericytes regulate cerebral blood flow, promote vascular stability, and remove toxic cell by-products from the CNS [45,46].

### 2.4. Basement Membrane

The BM, a distinct type of extracellular matrix (ECM), is primarily located beneath endothelial and epithelial cells in the brain, with pericytes acting as the separating barrier (Figure 1A) [47,48]. The BM serves various functions, including providing structural support, anchoring cells, and facilitating signal transduction [49,50,51]. These components primarily consist of type IV collagen, laminin, nidogen, perlecan, and other structural proteins. BMECs, pericytes, and astrocytes on the BBB primarily synthesize these ECM proteins [52,53]. These ECM structural proteins form the fundamental physical framework that supports TJ assembly in cells [54]. Signal transduction between the BM and its associated cells primarily relies on two types of matrix transmembrane receptors: dystroglycan and integrins. It is also closely associated with extracellular ligands of the ECM [50]. The binding of these receptors or ligands can activate multiple growth factors and signaling cascades, regulating cell growth, differentiation, migration, and survival during the development and maintenance of the BBB. During cerebrovascular formation, angiogenic endothelial cells (ECs) express α4β1 and α5β1 integrins, which bind to the ECM ligand fibronectin and induce cell proliferation through MAPK (Mitogen-activated protein kinase) signal transduction [55,56].

In summary, the BBB consists of four primary physiological barrier functions. The first function is the physical barrier, which is formed by TJs between adjacent endothelial cells. This physical barrier is characterized by the absence of intercellular clefts and low pinocytosis activity, effectively preventing the passive absorption of endogenous and exogenous compounds by the brain. The second barrier function is the biochemical barrier, which is formed by numerous inflow and outflow transporters located on the lumen and adventitia of endothelial cells [12,57]. These transporters tightly regulate the entry and exit of substances into the brain, actively limiting the brain’s absorption of foreign organisms and regulating nutrient supply. The third barrier function is the metabolic barrier, which is formed by a variety of metabolic enzymes present in endothelial cells [58]. This barrier function is responsible for the degradation of neurotoxins, drugs, and other compounds. The fourth barrier function is the immune barrier, which is composed of BMECs and regulated by astrocytes. This barrier function restricts the entry of immune cells from the periphery into the brain.

## 3. The Relationship between the Blood–Brain Barrier and AD Pathology

Dysfunction of the BBB/NVU can be observed in three main pathological aspects: (1) Leakage of circulating substances from plasma to the CNS; (2) Dysregulation of transporters leading to inadequate nutrient supply, accumulation of toxins in the CNS, or entry of other compounds into the brain; (3) Abnormal expression or secretion of cell proteins in the BBB/NVU, which promotes inflammation, oxidative stress, and neuronal damage [59]. Current evidence [60,61,62] indicates that dysfunctional BBB/NVU cells contribute to the development of various CNS disorders, including AD, which is characterized by neurodegeneration and cognitive decline.

**Figure 2:**
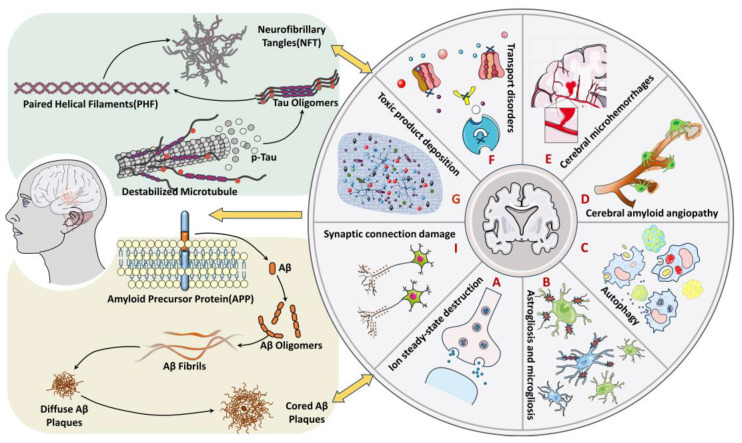
The connection between Alzheimer’s disease pathology and blood–brain barrier damage. The pathology of Alzheimer’s disease is closely related to the disruption of the blood–brain barrier; Aβ deposition and neurofibrillary tangles, as key pathological participants, interact with the blood–brain barrier. Firstly, under AD pathology, Aβ plaque deposition is caused by the gradual accumulation of Aβ monomers formed by the cleavage of APP (Aβ Oligomers, Aβ Fibrils, Diffuse Aβ Plaques), and NFTs are formed by the increased dissociation and hyperphosphorylation of tau proteins in the unstable microtubule system (p-tau, tau oligomers, paired helical filaments). Subsequently, Aβ and NFTs affect the blood–brain barrier, causing structural and functional disorders of the blood–brain barrier, leading to the following: (**A**) Ion steady-state destruction; (**B**) Astrogliosis and microgliosis; (**C**) Autophagy; (**D**) Cerebral amyloid angiopathy; (**E**) Cerebral microhemorrhages; (**F**) Transport disorders; (**G**): Toxic product deposition; (**I**) Synaptic connection damage. Conversely, related damage to the blood–brain barrier also promotes the formation of Aβ and NFTs. Finally, they jointly promote the pathological development of AD in this cycle.

**Figure 3:**
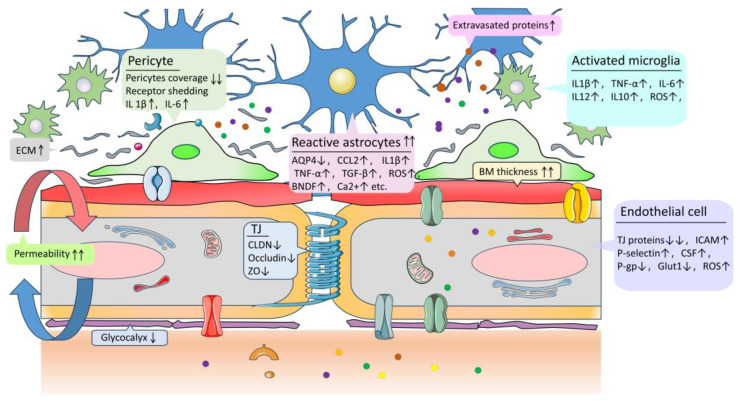
The specific manifestations of BBB in the AD process. The changes in the main components of the blood–brain barrier are closely associated with the progression of Alzheimer’s disease (AD). Dysregulation of endothelial cell transport proteins, activation of astrocytes and microglia promoting neuroinflammation, as well as oxidative stress reactions lead to decreased coverage of pericytes and subsequent receptor shedding, alterations in the glycocalyx and basement membrane, decreased neuronal cell adhesion, decreased tight junction proteins, increased blood–brain barrier permeability, and increased protein extravasation. All of these are specific pathological manifestations of the blood–brain barrier during the progression of AD. CLDN, claudin; ZO, zona occludens; ICAM, intracellular adhesion molecules; CSF, cerebral spinal fluid; P-gp, P-glycoprotein; Glut1, glucose transporter 1; ↓:downregulation or inhibition; ↓↓: more severe downregulation; ↑: upregulation or activation; ↑↑: more severe activation.

**Figure 4:**
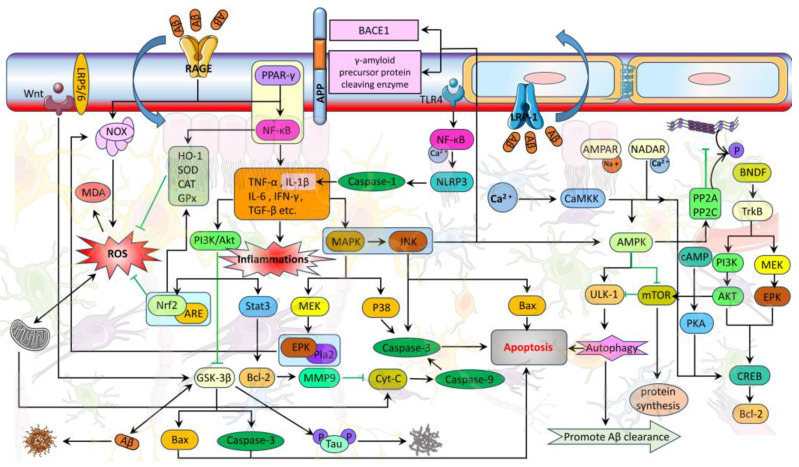
Signaling pathways associated with BBB disruption in AD. BBB disruption in AD leads to dysregulation of multiple signaling pathways. These dysregulated pathways include the inflammatory response, oxidative stress, neurotransmitter imbalance, and cellular apoptosis. The dysregulation of these signaling pathways may contribute to neuronal damage and cognitive decline.

The pathogenesis of AD is associated with structural changes and dysfunctions of the BBB. These dysfunctions include reduced endothelial transport, loss of TJ integrity, and degeneration of pericytes and astrocytes, which can lead to increased BBB permeability, microhemorrhages, impaired glucose transport, impaired P-glycoprotein function, perivascular deposition of blood-borne products, disruption of ionic homeostasis, altered signaling, and immune infiltration (Figure 2 and Figure 3). These changes ultimately result in neuronal dysfunction and neurodegeneration [5,12,60]. Additionally, BBB dysfunction triggers neuroinflammation and oxidative stress, which enhance the activity of β-amyloid precursor protein-cleaving enzyme 1 (BACE1) and γ-amyloid precursor protein-cleaving enzyme, ultimately promoting the production of Aβ (Figure 2 and Figure 4) [63]. At the same time, the decrease in the level of low-density lipoprotein receptor-related protein 1 (LRP-1) and the increase in the level of the receptor for advanced glycation endproducts (RAGE) in the BBB impairs the transport of Aβ from the brain to the peripheral circulation, leading to its accumulation in the brain. LRP-1, a member of the low-density lipoprotein (LDL) receptor family, functions as a versatile scavenger and cargo transporter with signal transduction activity [64]. RAGE, a receptor for advanced glycation end products, is a multiligand receptor of the immunoglobulin superfamily located on the cell surface. RAGE expressed on the luminal side of the BBB facilitates the transport of Aβ from the blood to the brain. The progressive accumulation of Aβ in the brain, along with BBB dysfunction, can create a feedback loop that contributes to cognitive impairment and dementia (Figure 2 and Figure 4) [9]. Furthermore, BBB dysfunction interacts with tau pathology by inducing hyperphosphorylation of the tau protein, leading to the formation of NFTs. Additionally, certain tau proteins can cross the BBB in both directions, exacerbating tau pathology and contributing to BBB damage (Figure 2 and Figure 4) [9,65,66,67,68].

The accumulation of Aβ and tau in the brain is widely recognized as a typical pathological marker of AD. Their interaction with the BBB leads to various pathological changes, which can be categorized into the following eight parts: (1) Dysregulation of calcium ions in neurons and astrocytes leads to excitotoxicity and astrocytosis (Figure 2A) [69]. (2) The increase in reactive astrocytes and microglia affects synaptic transmission (Figure 2B) [70,71]. (3) The decrease in lysosomal degradation ability of neurons leads to autophagy and neuronal loss (Figure 2C) [72]. (4) The accumulation of Aβ in cerebral vessels promotes the development of cerebral amyloid angiopathy (CAA). This process also results in the rupture of the vascular wall, leading to cerebral microbleeds (Figure 2E), infarction, white matter lesions, and ultimately the destruction of synapses and neurovascular networks [73]. (5) CAA causes neurovascular dysfunction (Figure 2D), which further impairs the cognitive function of AD patients [74,75]. (6) Myelin breakdown affects synaptic connections in AD (Figure 2I) [76]. (7) The detachment or inactivation of receptor and enzyme-active substances leads to the failure of signal transduction and substance transport (Figure 2F). (8) The deposition of blood-derived products and neurotoxic substances leads to neuronal degeneration (Figure 2G).

## 4. The Specific Mechanism of BBB Affecting AD Progression

### 4.1. Endothelial Cells and Tight Junction Damage

In AD, the accumulation of Aβ leads to structural changes in BMECs, resulting in the disruption of cell-to-cell connections and impairing their interaction with other elements of the NVU. This directly affects the permeability of the BBB [77,78]. Additionally, the expression of TJ proteins, such as zonula occludens protein 1 (ZO-1), occludin, and claudin-1, -3, and -5, is decreased. Platelet endothelial cell adhesion molecule-1 (PECAM-1/CD31) shedding and endothelial cell apoptosis also occur (Figure 3) [79,80,81]. The disruption and remodeling of TJ expose various mechanisms of BMEC injury. For example, Aβ induces the activation of the Ca^2+^/calmodulin-activated phosphatase calcineurin, leading to cytotoxic effects. Inflammatory mediators, including cytokines (e.g., IL-1β, IL-6, TNF-α, IL-12, and IL-23), chemokines (e.g., CCL2, CCL4, CCL3, CCL7, and CXCL10), complement components (e.g., C1q and C3), adhesion molecules (e.g., intercellular adhesion molecule (ICAM), p-selectin, and e-selectin), and matrix metalloproteinases (MMPs) of TJs, are activated in BMECs (Figure 3).

Simultaneously, leukocytes, microglia, and perivascular macrophages are collectively attracted to the perivascular region, along with mitochondrial damage and an increase in reactive oxygen species, such as NADPH (nicotinamide adenine dinucleotide phosphate) oxidase-2. These mechanisms described above contribute to the toxicity of endothelial Aβ and play a role in the inflammatory response of BMECs to TJ remodeling [82,83,84,85]. Additionally, endothelial cells enhance the transcellular transport of blood-derived substances through processes such as large protein endocytosis, gelatinase-dependent endocytosis, and gap-mediated transcytosis or vesicle transport [86,87,88,89]. The augmentation of paracellular and transcellular pathways results in the leakage of the NVU in AD. Despite limited evidence regarding histone modification, DNA methylation, and histone deacetylase activity in BMECs, recent studies [90,91] have demonstrated that dysregulation of microRNAs and long non-coding RNAs disrupts the integrity of the BBB. Specifically, the dysregulation of miR124, miR-107, and LINC00662 alters the transcription of ZO-1, claudin-5, and occludin in BMECs of AD patients through the modification of members belonging to the erythroid transformation-specific (ETS) transcription factor family and ETS-related genes [92].

### 4.2. Changes in Astrocyte Function and Structure

An increase in pathologic Aβ leads to functional and morphological changes in glial cells, particularly astrocytes and microglia, which are key cells involved in the inflammatory and immune response in the CNS. Aβ activates various receptors in astrocytes, with RAGE being the primary receptor that triggers the inflammatory pathway nuclear factor kappaB (NF-κB). This pathway is responsible for the transcription of numerous pro-inflammatory cytokines and chemokines in astrocytes, playing a crucial role in both pro-inflammatory (neurotoxicity) and immune regulation (neuroprotection) [93]. Additionally, NF-κB regulates other functions, including neuronal development, differentiation, apoptosis, neurite growth, and synaptic remodeling, all of which are dysregulated in AD [94]. Astrocytes produce cytokines such as TNF-α, IL-1β, IFN-γ, L-6, and TGF-β (Figure 3), which can enhance the activity of β- and γ-secretase enzymes through the c-Jun N-terminal kinase (JNK)-dependent MAPK pathway. This pathway cleaves APP and initiates Aβ formation [95].

Moreover, inflammatory mediators such as bradykinin can elevate intracellular calcium ion levels (Figure 3) through nicotinic receptors and the phosphoinositide 3-kinase (PI3K)-protein kinase B (Akt) pathway in astrocytes (Figure 4) [96]. Simultaneously, the interaction between Aβ and multiple receptors and neurotransmitters in astrocytic calcium ion dysregulation can account for neurotoxicity and neurodegeneration [94]. Astrocytic metabolic dysfunction may contribute to cognitive impairment in AD through the disruption of the glutamate and gamma-aminobutyric acid (GABA) glutamine cycle [97,98,99]. Parameshwaran et al. [100] demonstrated the involvement of N-methyl-D-aspartic acid (NMDA) and alpha-amino-3-hydroxy-5-methyl-4-isoxazole propionic acid (AMPA) receptors in the pathophysiology of AD. Additionally, dysfunction of NMDA receptors in astrocytes induced by Aβ can disrupt neuron–glial signaling [101]. Overall, dysfunction in glutamatergic signaling and excitatory toxicity can impact cognition. Oxidative stress in AD is strongly associated with mitochondrial dysfunction. The elevated levels of reactive oxygen species (ROS) and RNS result in the excessive production of superoxide (Figure 3), ultimately causing synaptic damage [102,103,104,105].

Aβ1-42 oligomers bind to RAGE on astrocytes, activating the NADPH oxidase (NOX) complex and leading to the production of ROS [106]. In addition, astrocytes can independently activate the extracellular signal-regulated kinase 1/2 (ERK 1/2) pathway and phosphorylate cytoplasmic phospholipase A2, resulting in mitochondrial dysfunction through the reduction of mitochondrial membrane potential, increased NOX activity, and excessive production of ROS [107]. Astrocytes express and secrete multiple MMPs, such as MMP-2 and MMP-9, which are capable of degrading both monomeric and fibroblastic forms of Aβ [101]. Structural changes in astrocytes result in the degeneration and disintegration of their protrusions, characterized by bead-like formations. These changes are accompanied by cytoplasmic vacuolization, atrophy, and swelling of astrocyte end pods [108,109]. At the end foot, the reduced levels of anchoring proteins, including AQP4, inward rectifier potassium channel (Kir4.1), and dystrophin 1, contribute to the further restriction of astrocyte–endothelial connections (Figure 3) [109,110,111]. These structural changes diminish the anchoring of astrocytes in the NVU and impair the interaction between cerebral blood flow and neurovascular coupling [112].

### 4.3. Pericyte Degradation

Pericyte degradation also plays a crucial role in the development of AD, as it affects the permeability of the BBB [113,114]. The deposition of vascular amyloid fibrils leads to the atrophy and invasive degradation of pericytes, resulting in reduced pericyte coverage (Figure 3). The extensive shedding of cell surface receptors expressed by pericytes causes them to lose function, leading to chronic hypoxia and decreased cerebral capillary perfusion, cerebral blood flow, and neurovascular coupling. These factors ultimately impact the integrity of the BBB [114,115,116]. The disruption of pericyte and endothelial cell contacts destabilizes TJs, while bidirectional pericyte/endothelial cell inflammatory signaling can also affect the stability of the TJ complex. Notably, inflammatory vesicle-forming, pattern-recognition receptor NLRP3 (nucleotide-binding domain-like receptor protein 3) and IL-1β are examples of factors that influence pericytes during BBB disruption (Figure 3) [114]. Furthermore, lower levels of pericyte-specific soluble proteins are directly associated with insufficient episodic memory, semantic memory, perceptual speed, visuospatial ability, and overall cognitive scores in AD patients [117].

### 4.4. Basement Membrane Thickening and Extracellular Matrix Protein Disorders

Thickening of the BM and remodeling of the ECM are crucial in the alteration of the NVU in AD vasculopathy. Previous studies have demonstrated a close association between BM thickening, fragmentation, and the presence of collagen and aggrecan in AD [80,118,119,120]. Moreover, the pathological accumulation of collagen can disrupt BBB transport, impair the interaction between endothelial cells and pericytes, and induce endothelial cell hypoxia, ultimately compromising BBB integrity [120]. Recent clinical and experimental studies have revealed elevated levels of perlecan, agrin, and fibronectin, as well as reduced levels of laminin, at the BBB/NVU in AD patients. The increase in these proteins in the perivascular space and BM is associated with Aβ accumulation, inflammation, apoptosis, and angiogenesis [120,121]. Indirectly, perlecan and fibronectin impact BBB integrity by promoting the inflammatory process and influencing intercellular interactions [121]. Alterations in the structure and composition of the BM ECM protein may facilitate the migration of circulating leukocytes to the brain during inflammation [59]. Changes in the glycocalyx and BM (Figure 3) affect the cell adhesion of NVU in AD. Research has demonstrated that these alterations are attributed to elevated levels of proteoglycans within the brain. Notably, hyaluronic acid, a glycocalyx and capillary BM proteoglycan, exhibits increased levels in cerebrospinal fluid samples of individuals with AD [122,123]. The rise in HA protein levels correlates with heightened cognitive decline in patients with AD [124]. Collagen and aggregation proteins, acting as proteoglycans, are also implicated.

## 5. The Related Pathways of Natural Plant Molecules Regulating the AD Blood–Brain Barrier

**Figure 5:**
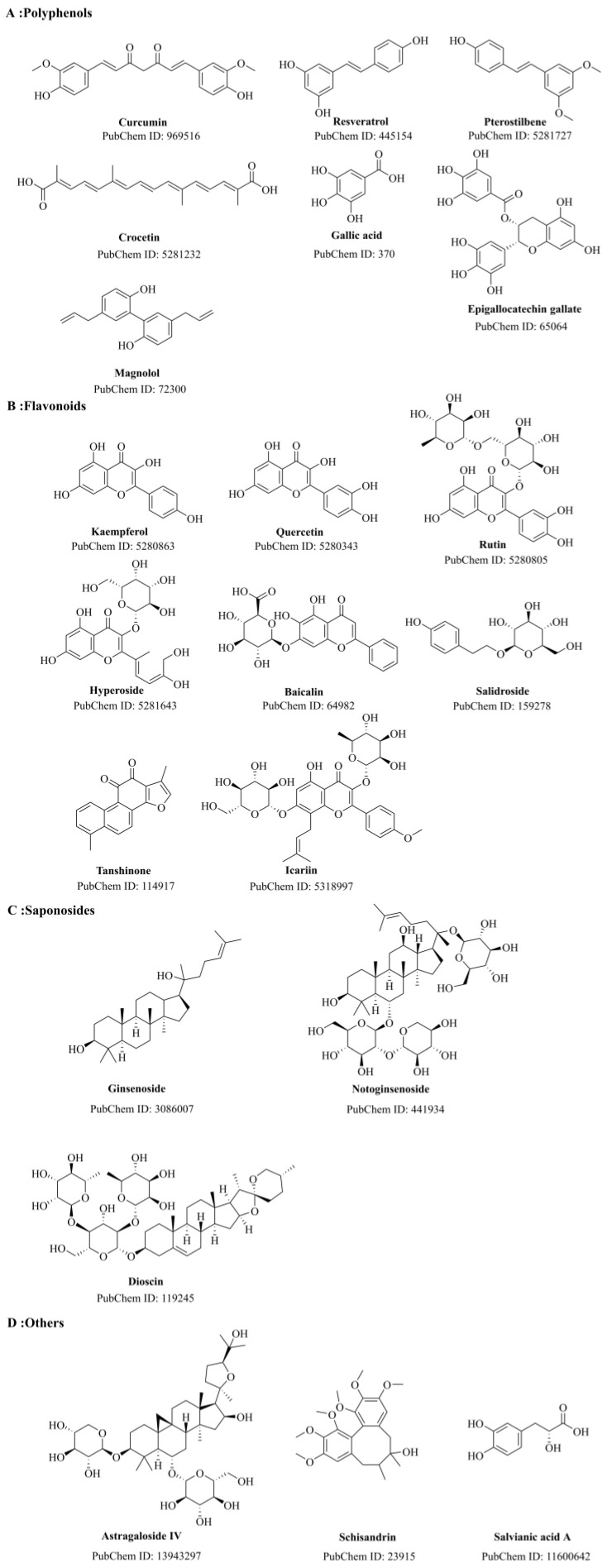
Chemical structure of these natural plant molecules.

This review initially examines the most recent advancements in the mechanism of BBB impairment in AD. Subsequently, we summarize and analyze experimental studies, recent reviews, and the literature on network pharmacology analysis pertaining to the utilization of natural plant molecules for the treatment of AD within the last decade. We summarize the effectiveness and mechanisms by which natural plant molecules regulate the BBB, with the corresponding pathways depicted in Figure 4. The chemical structures of these plant compounds are illustrated in Figure 5.

### 5.1. Polyphenols (Table 1)

#### 5.1.1. Curcumin

Curcumin, a natural compound primarily derived from the rhizomes of turmeric, Curcuma, Zedoary, and Acorus calamus, possesses potent anti-inflammatory and anticancer properties. It can also have a beneficial effect on neurovascular degeneration by protecting blood vessels [125]. Numerous studies [126,127,128,129] have demonstrated that curcumin can prevent the aggregation of Aβ, thereby reducing its formation. It has the ability to cross the BBB, alleviate inflammation, reduce oxidative damage, protect neurons, and decrease neurotoxicity in the brain. In a recent study involving APP/PS1 transgenic mice and primary rat mixed neuron/glia cultures [130], it was reported that curcumin not only attenuates the inflammatory response of microglia and astrocytes but also improves spatial memory deficits and promotes cholinergic neuronal function. These effects are achieved by regulating the activity of peroxisome proliferator-activated receptor gamma (PPARγ) and inhibiting the NF-κB signaling pathway in these cells. Wu X and colleagues analyzed the mechanism and targets of curcumin in treating AD using network pharmacology, and the results suggest that curcumin inhibits NF-κB transcription and protein levels, thereby suppressing TNF-α, IL-1β, cyclooxygenase-2 (COX-2), and inducible nitric oxide synthase (iNOS), reducing inflammation and cell apoptosis and ultimately decreasing Aβ deposition [131]. Additionally, curcumin has the ability to inhibit Aβ-induced tau hyperphosphorylation through the phosphatase and tensin homolog (PTEN)/Akt/Glycogen synthase kinase-3β (GSK-3β) pathway, showing great potential in improving targeted drug delivery and neuronal function recovery in the treatment of AD. Other studies have suggested that curcumin is a potent preventive drug for AD. It has been shown to protect cells from Aβ toxicity and prevent mitochondrial and synaptic toxicity in AD neurons induced by Aβ [132].

#### 5.1.2. Resveratrol

Resveratrol, a natural polyphenolic plant antitoxin, is primarily extracted from the roots and rhizomes of Japanese knotweed. In the plant, resveratrol mainly exists in the form of polydatin. Various animal models have confirmed the ability of resveratrol to protect BBB integrity and alter Aβ homeostasis [133,134]. In a randomized controlled trial of resveratrol treatment for AD, the protective effect of resveratrol on the BBB was further confirmed. The mechanism involves the regulation of MMP-9 to maintain BBB integrity, modulation of neuroinflammation, induction of adaptive immune responses, alteration of amyloid protein deposition, and restoration of brain Aβ homeostasis [135]. Resveratrol not only helps protect OVX+ D-galactosamine (D-gal) rats from Aβ42-5-mediated neuroinflammation by reducing NF-κB expression but also protects BBB integrity by increasing Claudin-5 and reducing RAGE and MMP-9 [136]. Additionally, research has found that resveratrol can reduce RAGE expression in vascular cells and increase LRP1 protein expression [137,138]. However, further large-scale studies are needed to confirm the specific mechanisms, and the issue of resveratrol’s low oral bioavailability needs to be addressed.

#### 5.1.3. Pterostilbene

Pterostilbene (PTS), a neuroprotective analog of resveratrol, exhibits greater BBB permeability and oral bioavailability compared to resveratrol. Originally derived from sandalwood, it has also been discovered in fruits such as blueberries and grapes, where it contributes to neuronal function and cognition during the aging process [139,140]. Furthermore, it can protect vascular endothelial cells by promoting macroautophagy and improving endothelial aging [141]. Studies have also reported that Schisandra chinensis extract inhibits vascular smooth muscle cell proliferation by downregulating MMP-2 through the MAPK pathway [142]. Li Q et al. [143] conducted a study on the biological mechanisms of PTS in the progression of AD. Their findings revealed that PTS can mitigate the accumulation of Aβ1-42 and hyperphosphorylation of tau, while inhibiting oxidative stress and neuroinflammatory responses in the hippocampus. Additionally, PTS reduces neuronal death by increasing the expression of b-cell lymphoma-2 (Bcl-2) and decreasing the levels of caspase-3 and BCL-2-associated X (Bax) in the hippocampus. These mechanisms were supported by measured biomarkers, including reduced levels of ROS and malondialdehyde (MDA), increased levels of superoxide dismutase (SOD) and glutathione (GSH), and suppressed levels of TNF-α, IL-1β, IL-6, and p-NF-κB. Previous studies have also demonstrated the anti-inflammatory effects of PTS and its ability to alleviate microglial neuroinflammatory responses induced by Aβ1-42 through inhibition of the NLRP3/caspase-1 inflammasome pathway [144]. Moreover, Meng et al. [145] discovered that PTS reduces dendritic spine density in APP/PS1 mice and increases the expression of vasodilator-stimulated phosphoprotein phosphorylation (pVASP), phosphorylated cAMP (cyclic adenosine monophosphate) response element-binding protein (pCREB), BDNF, and postsynaptic density protein 95 (PSD95). This suggests that PTS protects neurons from Aβ-induced neurotoxicity and cognitive impairment by regulating the PDE4A-CREB-BDNF pathway, as it reverses Aβ-induced cyclic AMP levels. Xu et al. [146] investigated the mechanism by which PTS affects Aβ1-42-induced cognitive impairment in mice and demonstrated that PTS effectively inhibits oxidative stress through the nuclear factor erythroid 2-related factor 2 (Nrf2) signaling pathway, a key regulator of oxidative homeostasis closely associated with oxidative stress responses.

#### 5.1.4. Crocetin

Saffron contains the active ingredients crocetin and crocin. A pharmacological review on the treatment of neurodegenerative diseases with crocetin [147] suggests that crocetin possesses antioxidant and anti-inflammatory properties and can improve mitochondrial dysfunction for neuroprotection against neurodegenerative diseases. In addition, saffron extract has been found to increase local cerebral blood flow and promote neuronal differentiation in an ischemic stroke rat model [148]. Bie X et al. [149] have also found that saffron extract’s protective effect on brain injury may be related to its ability to inhibit cell apoptosis and promote angiogenesis. In a study conducted by Abubakar Wani [150], the autophagy-inducing effect of crocetin was investigated using a 5XFAD mouse model of AD. The study found that crocetin can cross the BBB and induce autophagy via the Adenosine 5′-monophosphate (AMP)-activated protein kinase (AMPK) pathway, thereby facilitating the clearance of Aβ amyloid protein. Additionally, a separate study [151] demonstrated that treatment with crocetin can protect neurons and inhibit the activation of microglial cells, thereby preventing damage induced by lysophosphatidyl choline (LPC). In a study conducted by Zhang J et al. [152], crocetin was orally administered to an AD mouse model. The results demonstrated that crocetin significantly reduced pro-inflammatory cytokines in the plasma, enhanced anti-inflammatory cytokines, inhibited the activation of NF-κB, suppressed the expression of the tumor-suppressor protein p53 in the hippocampus, reduced Aβ levels in the brain regions, and improved deficits in learning and memory. A cell experiment [153] revealed that crocetin can enhance the production of ROS, decrease mitochondrial membrane potential, and reduce the phosphorylation of ERK. These findings provide evidence for the neuroprotective effect of crocetin against Aβ1-42-induced damage in mouse hippocampal neuronal cells (Ht22 cells), potentially attributed to its antioxidant properties.

#### 5.1.5. Gallic Acid

Gallic acid (GA) is a polyphenolic organic compound that is commonly found in various plants, including rhubarb, eucalyptus, cornus, peony, and rose. It has a wide range of applications in the medical field. Previous studies [154,155] have found that gallic acid not only has antioxidant capacity but also inhibits angiogenesis by suppressing vascular endothelial growth factor (VEGF) and adhesion molecules. Recently, in a study conducted by Mori et al. [156], the effects of GA were examined in an APP/PS1 transgenic AD mouse model. The researchers found that GA can reduce the deposition of β-amyloid protein in the brain parenchyma and blood vessels. Additionally, GA was found to enhance the activity of A disintegrin and metalloprotease 10 (ADAM10) and inhibit the activity of BACE1, which promotes the production of non-amyloid proteins and APP processing. This suggests that GA acts as a dual regulator of α/β secretase activity. Hajipour and Yu [157,158] conducted studies to observe the effects of GA on long-term potentiation (LTP) and histological changes in the hippocampus of Aβ-induced AD animal models. The results showed a significant increase in hippocampal LTP and an increase in the expression of synaptic marker proteins. GA was also found to reduce neuronal damage and cerebral amyloid neuropathy by scavenging free radicals and inhibiting the oligomerization of Aβ.

#### 5.1.6. Epigallocatechin Gallate

Epigallocatechin gallate (EGCG) is a bioactive compound derived from Camellia sinensis, serving as the principal constituent and the most prevalent entity within the catechin group [159]. Due to its unique chemical structure (Figure 5), EGCG exhibits potent antioxidant activity and prevents neurodegenerative diseases through anti-inflammatory mechanisms. Additionally, EGCG has been shown to improve vascular function by regulating endothelial nitric oxide synthase (eNOS) through its antioxidant activity [160]. A recent study [161] reviewed reports on EGCG and suggested that EGCG-induced autophagy may promote neuronal recovery and anti-aging functions in AD. Firstly, it induces autophagy to reduce protein aggregation caused by Aβ. Secondly, the antioxidant properties of EGCG may hinder ROS associated with mitochondrial dysfunction, utilizing autophagy as a mechanism for ROS clearance. Lastly, promoting autophagy can reduce the activity of pro-inflammatory cytokines/chemokines caused by chronic neuroinflammation induced by overactive glial cells. Furthermore, a study by Zhong et al. [162] found that EGCG inhibits the activation of typical NLRP3 and atypical caspase-11-dependent inflammasomes in primary rat microglia and the hippocampus of APP/PS1 mice induced by lipopolysaccharide (LPS) + Aβ through the toll-like receptor 4 (TLR4)/NF-κB pathway, reducing microglial inflammation and neurotoxicity. Another study by Nan et al. [163] demonstrated that EGCG can reduce the hyperphosphorylation of tau proteins, downregulate BACE1 and Aβ1-42 expression, and improve the antioxidant capacity, learning, and memory functions in AD rats. Recent research indicates that EGCG has the ability to cross the BBB. The interaction between polyphenols and Aβ may be facilitated by metal chelation, hydrogen bonding, or van der Waals forces, resulting in a reduction in the toxicity of Aβ oligomers and fibers. This reduction leads to a decrease in Aβ plaques in the brains of APP/PS1 mice, ultimately protecting neurons from damage [164].

#### 5.1.7. Magnolol

Magnolol, a bioactive compound found in Magnolia officinalis, has the ability to readily cross the BBB. Moreover, due to the synergistic effects of its antioxidant and anti-inflammatory properties, it can inhibit vascular smooth muscle cell proliferation through relevant pathways, thereby reducing vascular remodeling [165]. Zhu S et al. [166] propose that magnolol primarily safeguards the nervous system against brain diseases by protecting neurons and BMECs. They summarize the five functions of magnolol in neolignans as follows: (1) Enhancing neuronal function through the regulation of neurotransmitters and their receptors; (2) Reducing neurotoxicity by decreasing levels of APP, γ-secretase, and BACE1; (3) Inhibiting neuronal apoptosis by reducing Bax, caspase-3, and other markers of cell apoptosis; (4) Exerting anti-neuroinflammatory effects via the PPARγ/NF-κB pathway; (5) Exhibiting antioxidant effects through the MAPK and PI3K/Akt pathways, including the p38/MAPK and MAPK/ERK pathways. For BMECs, magnolol has been shown to enhance blood supply, normalize blood sugar levels, and improve their connectivity. Wang X [167] has made significant advancements in the study of inhibiting cell apoptosis. Magnolol has been found to inhibit cell apoptosis in APP/PS1 mice and Aβ-induced cell models by downregulating cleaved caspase-9 and Bax, while upregulating Bcl-2. Its mechanism is to promote autophagy by activating the AMPK/mammalian target of rapamycin (mTOR)/unc-51-like autophagy-activating kinase 1 (ULK1) pathway, degrading p62/sequestosome 1 (SQSTM1), and upregulating microtubule-associated proteins light chain 3 II (LC3 II) and Beclin-1 expression. This inhibits cell apoptosis and improves symptoms related to AD. Another study confirmed that magnolol has anti-inflammatory and antioxidant effects. It regulates the PI3K/Akt/GSK-3β and NF-κB pathways, inhibits neuroinflammation, amyloidosis, synaptic dysfunction, and improves cognitive impairment [168]. Xie Z et al. [169] confirmed that magnolol improves pathological Aβ in transgenic Caenorhabditis elegans. Magnolol depends on PPARγ activity and induces the expression of LXR, ABCA1, and ApoE mRNA. This mediates the adequate lysosomal clearance of Aβ in microglia. Additionally, magnolol inhibits the mRNA expression of NF-κB and inflammatory cytokines. It also activates Nrf2-ARE and reduces the production of ROS. A recent study showed that magnolol upregulates the level of CHRM1, activates the cAMP/protein kinase A (PKA)/CREB pathway, and weakens Aβ-induced vitality inhibition, tau hyperphosphorylation, and neuronal apoptosis [170].

**Table 1:** Summary of recent studies on the role and mechanisms of polyphenolic natural plant molecules on AD BBB-related targets.

Natural Plant Molecules	Experimental Subjects	Possible Mechanisms	Target of Action	Reference
Curcumin	APP/PS1 mouse	Regulating PPARγ activity ↓ NF-κB signaling pathway	Astrocytes and endothelial cells	Liu 2016 [130]
Resveratrol	AD patients	↓MMP9	Endothelial cells	Moussa 2017 [135]
	OVX+D-gal rat	↓ NF-κB signaling pathway ↑ Claudin-5 ↓ RAGE, MMP-9	Astrocytes and endothelial cells	Zhao 2015 [136]
	APP/PS1 mouse	↑ LRP1 protein expression	Endothelial cells	Santos 2016 [138]
Pterostilbene	The immortalized murine microglia cell line BV-2	↓ NLRP3/caspase-1 inflammasome pathway	Astrocytes and microglia	Li 2018 [144]
	APP/PS1 mouse	↑ PDE4A-CREB-BDNF pathway	Endothelial cells and astrocytes	Meng 2019 [145]
	Aβ1-42 induced mice	↑ Nrf2 signaling pathway	Endothelial cells and microglia	Xu 2021 [146]
Crocetin	AD model of 5XFAD mice	↑ Autophagy via regulating AMPK pathway	Endothelial cells and microglia	Wani 2021 [150]
	LPC-treated primary mixed mesencephalic neuron/glial cultures	↓ Microglia activation	Microglia	Zang 2022 [151]
	APP/PS1 mouse	↓ NF-κB activation ↓ P53 expression	Astrocytes and microglia	Zhang 2018 [152]
	Mouse hippocampal Ht22 cells	↑ Antioxidant capacity	Endothelial cells, astrocytes, and microglia	Kong 2014 [153]
Gallic acid	APP/PS1 mouse	↑ ADAM10 ↓ BACE1 activity	Endothelial cells and microglia	Mori 2020 [156]
	APP/PS1 mouse	↓ Aβ aggregation ↑ synaptic strength ↓ inflammation ↓ intracellular calcium influx	Endothelial cells and microglia	Yu 2019 [158]
Epigallocatechin gallate	BV2 cells and APP/PS1 mice	↓ TLR4/NF-κB pathway	Endothelial cells and microglia	Zhong 2019 [161]
	Aβ25-35 induced AD rat	↓ Hyperphosphorylation of tau protein ↓ BACE1 and Aβ1-42 expression	Endothelial cells and microglia	Nan 2021 [162]
	APP/PS1 mouse	↓ ROS production ↓ Aβ aggregation	Endothelial cells and microglia	Chen 2020 [163]
Magnolol	APP/PS1 mouse	↑ Autophagy via regulating AMPK/mTOR/ULK1 pathway	Endothelial cells and microglia	Wang 2023 [166]
	TgCRND8 mice	Regulating PI3K/Akt/GSK-3β and NF-κB pathway	Microglia and astrocytes	Xian 2020 [167]
	Transgenic caenorhabditis elegans	Regulating PPAR-γ activity ↓ NF-κB and inflammatory cytokines ↓ ROS via Nrf2-ARE	Endothelial cells and microglia	Xie 2020 [168]
	Aβ-induced SH-SY1Y cells	Regulating the cAMP/PKA/CREB pathway	Endothelial cells and microglia	Zhu 2022 [169]

↓: downregulation or inhibition; ↑: upregulation or activation.

### 5.2. Flavonoids (Table 2)

#### 5.2.1. Kaempferol

Kaempferol, a flavonoid compound commonly found in plants, is primarily derived from the rhizomes of the ginger family plant Kaempferia. It possesses various properties, including antibacterial, anticancer, antioxidant, and anti-inflammatory effects, and is widely present in fruits and vegetables. Kaempferol can be found in tea, broccoli, apples, strawberries, and beans, and has garnered increasing attention in recent years. It has been reported that resveratrol can protect the intestinal vascular barrier by inhibiting the expression of vascular growth-related signals [171]. Dong X et al. [172] conducted a comprehensive evaluation of the neuroprotective effects of kaempferol in both in vivo and in vitro models of AD based on 12 reliable studies. Their findings suggest that kaempferol improves AD through its antioxidant, anti-inflammatory, anti-apoptotic, and anti-acetylcholinesterase mechanisms. Recent studies, such as the one conducted by Silva Dos Santos J et al. [173], have also confirmed these mechanisms and demonstrated that kaempferol regulates specific pathways involved in AD progression (such as NF-κB, p38MAPK, and AKT) and achieves neuroprotection by regulating BDNF. Yan T’s research [174] indicates that kaempferol exerts neuroprotective effects by alleviating oxidative stress and enhancing the BDNF/tropomyosin-related kinase B (TrkB)/CREB pathway in Aβ. The antioxidant capacity of kaempferol, as evidenced by its ability to increase SOD and GSH levels and reduce MDA, has been widely reported in previous studies [175,176]. Yang Y L et al.’s study [177] demonstrated that kaempferol increased the levels of tyrosine hydroxylase (TH) and PSD95 in the striatum of mice. It also inhibited the production of pro-inflammatory cytokines, reduced the levels of monocyte chemoattractant protein-1 (MCP-1), ICAM-1, and COX-2 in striatal tissue, protected the integrity of the BBB, and downregulated the high mobility group box protein 1 (HMGB1)/TLR4 pathway. In a recent molecular docking and network pharmacology study by Singh et al. [178], quercetin, kaempferol, and isorhamnetin, which are highly active compounds found in ginkgo leaves, were found to have significant inhibitory activity against acetylcholinesterase (AChE) and GSK3β. These compounds may act on multiple targets in the AD protein network.

#### 5.2.2. Quercetin

Quercetin, one of the most widely distributed natural flavonoid compounds, is abundant in nature. Due to its lipophilic nature, it can easily cross the BBB and prevent neurodegenerative diseases [179]. An earlier study has already demonstrated the impact of quercetin on BMECs. It can regulate redox imbalance, exhibit strong antioxidant activity, and enhance the integrity of the BBB by protecting the permeability and characteristic enzyme activity of Aβ1-40-treated BMECs. This effect is speculated to be related to the regulation of Aβ active transport across the BBB [180]. Recent research, based on clustering and pharmacokinetic analysis, has confirmed that quercetin plays a role in the peripheral clearance of Aβ [181]. Moreover, Li M T et al. [182] investigated the protective effects of quercetin on HBMECs, focusing on cell vitality, migration, angiogenesis, and apoptosis. They found that quercetin not only positively regulates these aspects but also activates the Kelch-1ike ECH-associated protein l (Keap1)/Nrf2 signaling pathway. Additionally, it reduces activating transcription factor 6/glucose-regulated protein 78 expression and increases Claudin-5 and Zonula occludens-1 expression, which enhance the level of BBB junction proteins, thereby maintaining BBB integrity. Claudin-5, which is highly expressed in BMEC, plays a role in the formation of TJ chains that regulate BBB permeability [182,183]. Zamanian MY et al. investigated the effects of quercetin targeting the Nrf2 signaling pathway on experimental models of AD. They found that quercetin improves cognitive function by reducing Aβ levels, enhancing antioxidant activity, and modulating Nrf2 levels in the brain [184]. However, due to its low bioavailability, quercetin has a disadvantage. To overcome this, Rifaai RA et al. utilized quercetin nanoparticles to treat AD model rats. The results demonstrated that quercetin effectively reduced neurodegenerative changes, decreased the formation of Aβ and NFT, and mitigated the destructive effects of aluminum chloride on hippocampal neurons at the molecular, cellular, and subcellular levels [185].

#### 5.2.3. Rutin

Rutin, also known as rutoside or vitamin P, is a glycoside composed of the flavonol aglycone quercetin and the disaccharide rutinose. It can be found in rue leaves, tobacco leaves, orange peels, tomatoes, buckwheat flowers, and sophora japonica. Rutin has been shown to possess anti-inflammatory, antioxidant, anti-allergic, and anti-platelet activation factor effects [186]. The mechanisms by which rutin intervenes in neurodegenerative diseases include reducing pro-inflammatory cytokines, improving antioxidant enzyme activity, activating MAPK cascade reactions, downregulating PD junction and pro-apoptotic gene mRNA expression, upregulating ion transport and anti-apoptotic genes, and restoring the activity of mitochondrial complex enzymes [187]. Additionally, rutin plays a role in neuron plasticity and survival in the CNS. It activates BDNF and MAPK cascades (ERK 1/2 and CREB), leading to a significant increase in the gene expression of ERK 1, cAMP response element-binding protein (CREB), and BDNF in the rat hippocampus [187,188]. Studies have demonstrated that rutin plays a regulatory role in tau hyperphosphorylation by increasing PP2A levels, thereby reducing tau levels in mice with AD. Additionally, rutin downregulates the NF-kB pathway, which inhibits neuroglial proliferation and neuroinflammation. It also prevents synapse phagocytosis via microglia and rescues synapse loss in the mouse brain, leading to a significant improvement in cognitive ability [189]. In a study conducted by Xu P X et al. [190], the effects of rutin on APP/PS1 transgenic mice were investigated. The results showed that oral administration of rutin effectively alleviated memory deficits in AD transgenic mice. Furthermore, it reduced levels of oligomeric Aβ, increased SOD activity, and improved the GSH/glutathione disulfide (GSSG) ratio. Rutin also decreased GSSG and MDA levels, downregulated microgliosis and astrogliosis, and reduced brain levels of IL-1β and IL-6. Bermejo-Bescós P et al. [191] obtained similar findings in their study, but they also discovered that rutin can reduce APP expression and BACE1 activity while increasing ADAM10 levels.

#### 5.2.4. Hyperoside

Hyperoside is widely distributed in various plants, with the highest concentration found in Hypericum perforatum. It exhibits multiple physiological activities, including anti-inflammatory, antispasmodic, diuretic, antitussive, protein assimilation, and cardiovascular system protection, rendering it an indispensable natural product. Chen L et al. [192] demonstrated that prolonged administration of hyperoside drugs enhances spatial learning and memory abilities in APP/PS1 transgenic mice, reduces amyloid plaque deposition and tau phosphorylation, and mitigates neuroinflammation and oxidative stress in the brains of APP/PS1 mice by downregulating BACE1 and GSK3β levels, thereby attenuating the activation of microglia and astrocytes. Subsequently, Yi J H et al. [193] discovered that hyperoside potentially enhances synaptic function and improves memory in AD model mice through the regulation of synaptic calcium-permeable AMPA receptors. Additionally, a recent study [194] revealed that hyperoside alleviates β-amyloid protein toxicity by modulating the endoplasmic reticulum (ER)-mitochondrial calcium-signaling cascade, thereby exerting anti-Aβ aggregation, BACE inhibition, and neuroprotective effects. This mechanism has been validated in both in vitro and in vivo models.

#### 5.2.5. Baicalin

Baicalin (BAI) is a flavonoid compound that has been isolated from the root of Scutellaria baicalensis. It has been extensively studied and proven to possess significant anti-inflammatory and neuroprotective properties. Furthermore, baicalin has been reported to relax vascular smooth muscle and lower blood pressure in spontaneously hypertensive rats [195]. BAI has been shown to effectively reduce microglial activation and the release of pro-inflammatory cytokines. It also inhibits neuronal apoptosis by suppressing the activation of the NLRP3 inflammasome and the TLR4/NF-κB signaling pathway. These mechanisms have been confirmed in both animal and cell experiments [196,197]. Xiong J et al. discovered that BAI can inhibit Aβ-induced microglial activation by regulating the Janus kinase 2/signal transducer and activator of the transcription-3 signaling pathway [198]. In cell experiments conducted by Song Z et al., it was found that BAI can regulate the progression and apoptosis of neuronal cell cycles in Aβ-treated human neuroblastoma cells (SH-SY5Y) by inhibiting the renin–angiotensin system-ERK signaling pathway [199]. Prior to this, Ding H et al. [200] studied the potential effects of BAI on Aβ toxicity in an AD rat model. BAI achieves its anti-apoptotic effects by regulating mitochondrial membrane potential, the Bax/Bcl-2 ratio, cytochrome-c (Cyt-C) release, and caspase-9/-3 activation. Additionally, BAI enhances antioxidant capacity by restoring the activity of antioxidant enzymes (SOD, catalase, and glutathione peroxidase) and upregulating their gene expression, which is also associated with Nrf2 activation. Recent research on mitochondria by Yu HY et al. [201] revealed that BAI mitigates memory deficits and mitochondrial fragmentation caused by amyloid β oligomers through the regulation of the PDE-PKA-Dynamin-related protein 1 (Drp1) signaling pathway. BAI can decrease PDE4 levels, enhance AD microtubule-associated protein-2 levels, and increase the expression of synaptic proteins and PSD95.

#### 5.2.6. Salidroside

Salidroside (Sal), the primary bioactive component derived from the root and stem of the medicinal plant Rhodiola rosea, exhibits diverse pharmacological effects, including anti-fatigue, anti-aging, immune regulation, and free-radical scavenging. Liu ZB et al. [202] have found that salidroside shows great potential in the treatment of peripheral artery diseases, as it may stimulate angiogenesis through autocrine, paracrine, and endothelial progenitor cell mechanisms, which is likely the result of a combination of multiple signaling pathways. A preliminary study [203] demonstrated that Sal alleviates D-gal-induced memory deficits and inflammation by upregulating Sirtuin 1 (SIRT1) to inhibit the NF-κB signaling pathway. Furthermore, Sal can decrease microglial cell activation and reduce the levels of pro-inflammatory factors IL-1β, IL-6, and TNF-α in the brain [204]. Cai Y et al. [205] investigated the potential mechanism of Sal in slowing the progression of AD and discovered that Sal can significantly ameliorate AD pathology by inhibiting the activation of NLRP3 inflammasomes and the release of downstream pro-inflammatory factors. This mechanism can function either through direct inhibition of NLRP3/Caspase-1 or indirect inhibition of the TLR4/NF-κB/NLRP3/Caspase-1 signaling pathway. Subsequent research demonstrated that Sal effectively inhibits Aβ-induced apoptosis in human pancreatic carcinoma cells (PC-2) by activating the ERK 1/2 and AKT signaling pathways [206]. Sal has another mechanism that protects neuronal synapses from damage, reduces Aβ levels, increases the expression of PSD95, NMDA receptor 1, and Ca^2+^/calmodulin-dependent protein kinase II, and upregulates the phosphatidylinositol peptide PI3K/Akt/mTOR signal [207]. Additionally, Yang S et al. [208] provided evidence that Sal inhibits neuronal ferroptosis in both AD model mice and HT22 cell experiments by activating the Nrf2/HO-1 (heme oxygenase 1) signaling pathway. Building on this, another study discovered that Sal directly binds to the transcription factor Nrf2, preventing its degradation by blocking its interaction with the ubiquitin ligase Keap1 and promoting Nrf2-mediated SIRT3 transcription. This pathway safeguards mitochondria and neuronal synapses, thus mitigating AD pathology [209]. The most recent study [210] demonstrates that Sal not only enhances iron metabolism and mitochondrial metabolism but also inhibits the infiltration of cytotoxic T lymphocytes (CD8+ T cells).

#### 5.2.7. Tanshinone

Tanshinone is a liposoluble phenanthrene quinone compound derived from the root and stem of Salvia miltiorrhiza. It possesses antibacterial, anti-inflammatory, and blood-activating effects, and has not been found to have significant toxic side effects. Tanshinone IIA has been shown to improve cognitive ability and reduce neuroinflammation by inhibiting the RAGE/NF-κB signaling pathway. It also alleviates the loss of synapse-related proteins (Syn and PSD95) and neurons [211]. Both Tanshinone IIA and cryptotanshinone can decrease the expression of the glial fibrillary acidic protein (GFAP), S-100 calcium-binding protein beta chain (S100β), COX-2, iNOS, and NF-kBp65 [212]. Additionally, Li J et al. [213] discovered that Tanshinone IIA can increase the levels of the neuron-specific nuclear protein (NeuN), Nissl bodies, and IκB. It inhibits the proliferation of astrocytes in the AD model, thereby exerting anti-inflammatory and neuroprotective effects. Tanshinone IIA activates the PI3K/Akt signaling pathway to inhibit GSK-3β, significantly reducing tau hyperphosphorylation, preventing neuron loss and apoptosis, and reversing cholinergic dysfunction and oxidative stress [214,215]. Furthermore, Tanshinone IIA promotes the clearance of AD-related proteins and activates the synthesis of synaptic BDNF [216]. Recent research has also focused on Tanshinone IIA’s ability to inhibit ER stress. He Y et al. [217] discovered that Tanshinone IIA not only reduces the deposition of Aβ plaques but also inhibits cell apoptosis caused by ER stress. This is achieved by inhibiting the activation of the JNK pathway, which leads to an upregulation of the Bcl-2/Bax ratio and a downregulation of caspase-3 protein activity. Wan C et al. [218] conducted research that revealed Tanshinone IIA administration increases LRP1 expression and reduces RAGE expression. Based on these findings, it can be inferred that Tanshinone IIA promotes Aβ transport by alleviating SIRT1-mediated ER stress in BMECs, thereby improving cognitive defects in APP/PS1 mice.

#### 5.2.8. Icariin

Icariin is the main active ingredient extracted from the dried stems and leaves of various species of Epimedium. It can increase blood flow in the cardiovascular and cerebrovascular systems and regulate immune function and bone metabolism. Additionally, it has the effects of tonifying the kidney and invigorating yang, as well as being anti-aging. It has a significant protective effect on the vascular system and can effectively regulate cellular autophagy. Research has shown that it can regulate autophagy to affect the angiogenic capacity of endothelial cells [219]. Previous studies have confirmed that ICA has multiple regulatory pathways in AD pathology, including the BACE1, NO/cyclic guanosine monophosphate (cGMP), Wnt/Ca^2+^, and PI3K/Akt signaling pathways. Additionally, the NF-κB, MAPK, ERK, and phospho-ERK/Eif2α signaling pathways may be associated with alleviating ER stress in AD mice. ICA can inhibit microglial cell activity by regulating PPARγ, transforming growth factor beta-activated kinase (TAK)/inhibitor of NF-kappaB kinase (IKK)/NF-κB, and JNK/p38 MAPK signaling pathways. It also protects neurons by reducing mitochondrial oxidative stress damage and improves synaptic structure by regulating PSD95 [220]. Recent research has provided further evidence and insights into the anti-inflammatory mechanism of ICA in AD. This mechanism involves the inhibition of pro-inflammatory cytokines, the upregulation of anti-inflammatory cytokines, the inhibition of the increase in M1 phenotype microglia in the hippocampus and PFC, and the enhancement of PPARγ expression in the hippocampus and PFC [221]. Lu Q et al. [222] discovered that ICA enhances the proliferation and differentiation of hippocampal neural stem cells exposed to Aβ through the BDNF-TrkB-ERK/Akt pathway. In terms of neuroprotective mechanisms, ICA downregulates the expression of BACE1, reduces the expression of cytotoxic Aβ1-42, and inhibits neuronal apoptosis by increasing the ratio of Bcl-2 to Bax [223]. Jiang X et al. [224] found that ICA improves the function of autophagosomes in an Aβ1-42-induced rat model experiment, which is associated with enhanced phosphorylation of PKB/AKT and p70S6K. Additionally, ICA prevents Aβ1-42-induced cell loss, mitochondrial damage, nuclear membrane abnormalities, and increased hippocampal cell nuclear chromatin aggregates. It also reduces the expression of caspase-3 cleavage, oxidative stress in the brain, and astrocyte activation. Another study [225] using the SAMP8 mouse model demonstrated that ICA reduces the formation of autophagosomes in the hippocampus, decreases the expression of LC3 II and p62 proteins, reduces the number of senescent cells in the brain, inhibits neuronal loss, and counteracts structural changes in neurons.

**Table 2:** Summary of recent studies on the role and mechanisms of flavonoid natural plant molecules on AD BBB-related targets.

Natural Plant Molecules	Experimental Subjects	Possible Mechanisms	Target of Action	Reference
Kaempferol	Aβ1-42-induced mice	↓ Oxidative stress ↑ BDNF/TrkB/CREB	Endothelial cells and microglia	Yan 2019 [174]
	STZ-induced AD rats	↑ SOD and GSH ↓ MDA	Endothelial cells and microglia	Babaei 2021 [175]
	STZ-induced AD rats	↑ SOD and GSH ↓ MDA and TNF-α	Endothelial cells and microglia	Kouhestani 2018 [176]
	LPS-induced mice	↓ Neuroinflammation ↑ BBB integrity ↓ HMGB1/TLR4 pathway	Endothelial cells and microglia	Yang 2019 [177]
Quercetin	Aβ damages cerebral microvascular endothelial cells	↓ Oxidative stress ↑ Transendothelial permeability regulating enzymes and maintaining BBB integrity	Endothelial cell	Li 2015 [180]
	Hypoxia-injured endothelial cells	Regulating Keap1/Nrf2 pathway ↑BBB junction proteins Maintaining BBB integrity	Endothelial cell	Li 2021 [182]
Rutin	Aβ-induced rat	Regulating BDNF and MAPK cascades	Astrocytes and microglia	Mogbelinejad 2014 [188]
	Male Tau-P301S mice	Regulating PP2A, NF-kB pathway	Endothelial cells and microglia	Sun 2021 [189]
	APP/PS1 mouse	↓ Oxidative stress ↓ Inflammatory response ↓ Aβ oligomer activity	Endothelial cells and microglia	Xu 2014 [190]
	Transgenic TgAPP mice	↓ APP expression ↓ BACE1 activity	Endothelial cells and microglia	Bermejo-Bescós 2023 [191]
Hyperoside	APP/PS1 mouse	↓ BACE1, GSK3β	Endothelial cells and microglia	Chen 2021 [192]
	Aβ-induced AD mice	Regulating synaptic calcium-permeable AMPA receptor	Endothelial cells	Yi 2022 [193]
	APP/PS1 mouse	↓ Aβ aggregation ↓ BACE1	Endothelial cells and astrocytes	Song 2023 [194]
Baicalin	APP/PS1 mouse and BV2 cells	↓ Activation of NLRP3 inflammasome ↓ TLR4/NF-κB signaling pathway	Microglia and astrocytes	Jin 2019 [196]
	Aβ1-42-induced mice	↓ Neuroinflammation	Microglia and astrocytes	Chen 2015 [197]
	Aβ-induced BV2 microglia	Regulating JAK2/STAT3 signaling pathway	Microglia	Xiong 2014 [198]
	Aβ induced SH-SY5Y cells	Regulating Ras-ERK signaling pathway	Microglia and astrocytes	Song 2022 [199]
	Aβ1-42 induced rat	Regulating Nrf2 pathway ↓ Oxidative stress	Endothelial cells and microglia	Ding 2015 [200]
	Aβ-induced AD mice	Regulating PDE-PKA-Drp1 signaling	Endothelial cells and microglia	Yu 2022 [201]
Salidroside	D-gal induced mice	↑ SIRT1 ↓ NF-κB pathway	Endothelial cells and astrocytes	Gao 2016 [203]
	SAMP8 mice	↓ Pro-inflammatory cytokines	Endothelial cells and astrocytes	Xie 2020 [204]
	Aβ1-42 induced mice and D-gal induced mice	↓ NLRP3 inflammasome-mediated pyroptosis	Microglia and astrocytes	Cai 2021 [205]
	PC-2 cells	Regulating ERK1/2 and AKT signaling pathways	Microglia and astrocytes	Liao 2019 [206]
	APP/PS1 mouse	↑ PSD95, NMDAR1 ↑ Calmodulin-dependent protein kinase II Regulating phosphatidylinositol PI3K/Akt/mTOR signaling	Endothelial cells, microglia, and astrocytes	Wang 2020 [207]
	Aβ1-42-induced mice and Glu-damaged HT22 cells	Regulating Nrf2/HO1 signaling pathway	Endothelial cells and microglia	Yang 2022 [208]
	AD model of 5XFAD mice	Regulating Nrf2/SIRT3 signaling pathway	Endothelial cells and microglia	Yao 2022 [209]
	SAMP8 mice	↑ Nrf2/GPX4 axis ↓ CD8+ T cell infiltration	Endothelial cells, microglia, and astrocytes	Yang 2023 [210]
Tanshinone	APP/PS1 mouse	Regulating RAGE/NF-κB signaling pathway	Endothelial cells and microglia	Ding 2020 [211]
	Aβ-induced AD mice	↓ Neuroinflammatory factors	Astrocytes and microglia	Maione 2018 [212]
	Aβ1-42-induced mice	↓ NF-κB ↑ NeuN, Nissl body, and IκB	Endothelial cells, astrocytes, and microglia	Li 2015 [213]
	Aβ1-42-induced rat	↓ The activity of ERK and GSK-3β	Microglia and astrocytes	Lin 2019 [214]
	APP/PS1 mouse	↑ PI3K/Akt pathway ↓ GSK-3β ↓ Tau hyperphosphorylation	Endothelial cells, microglia, and astrocytes	Peng 2022 [215]
	APP/PS1 mouse	↑ The clearance of AD-related proteins ↑ BDNF-TrkB pathway	Endothelial cells and astrocytes	Li 2016 [216]
	APP/PS1 mouse	↓ Aβ plaque deposition ↓ ER stress-induced apoptosis	Endothelial cells and microglia	He 2020 [217]
	APP/PS1 mouse	Regulating SIRT1 expression ↓ ER stress ↑ LRP1 ↓ RAGE	Endothelial cells and microglia	Wan 2023 [218]
Icariin	APP/PS1 mouse	↓ Pro-inflammatory cytokines ↑ PPARγ	Microglia and astrocytes	Wang 2019 [221]
	Hippocampal neural stem cells treated with Aβ25-35	Regulating BDNF-TrkB-ERK/Akt signaling pathway	Astrocytes and microglia	Lu 2020 [222]
	SAMP8 mice	↓ BACE1	Endothelial cells and microglia	Wu 2019 [223]
	Aβ1-42-induced rat	↑ Autophagic lysosomal function	Astrocytes and microglia	Jiang 2019 [224]
	SAMP8 mice	Regulating autophagy	Astrocytes and microglia	Chen 2019 [225]

↓: downregulation or inhibition; ↑: upregulation or activation.

### 5.3. Saponosides (Table 3)

#### 5.3.1. Ginsenosides

Ginseng, a highly valued medicinal herb renowned for its life-saving properties, holds great prestige in China. The primary active molecules of ginseng, known as ginsenosides, possess a wide range of pharmacological effects. These effects include anti-inflammatory, antioxidant, anti-apoptotic, inhibition of Ca^2+^ influx, and mitochondrial protection. These mechanisms have the ability to suppress excitotoxicity, regulate neurotrophic factors, promote neural regeneration, and reduce neural damage in neurological diseases, such as AD [226,227]. Ginsenosides have been found to inhibit the phosphorylation of tau proteins, the accumulation of Aβ1-42, and the expression of BACE1. Additionally, they exhibit potent antioxidant activity by regulating the expression of Nrf2, HO-1, and NQO1 (NAD(P)H-dependent Quinone Oxidoreductase 1) genes. Furthermore, they significantly increase the activity of SOD, Catalase (CAT), and glutathione peroxidase (GPx) [228,229]. Recent research has also confirmed their antioxidant properties, as they reduce the production of intracellular ROS and MDA, increase GSH and SOD levels, and restore the mitochondrial membrane potential of neuronal cells through activation of the AMPK/Nrf2 signaling pathway [230]. Conversely, ginsenosides inhibit apoptosis by regulating the Wnt/GSK-3β/β-catenin signaling pathway, increasing the Bcl-2/Bax ratio, promoting the expression of neuronal marker microtubule-associated protein 2 (MAP2) and NeuN, and inhibiting the expression of p53, Cyt-C, Caspase-3, Caspase-9, GSK-3β, and β-catenin [228,229]. Quan Q et al.’s research [231] suggests that ginsenosides can reduce the expression of cyclin-dependent kinase 5 (CDK5) and inhibit the phosphorylation of serine 273 in PPARγ. A recent study conducted by Li X et al. [232] discovered that ginsenosides can inhibit the NLRP1 inflammasome and improve autophagy dysfunction. This inhibition leads to a decrease in the expression levels of NLRP1, Caspase-1, IL-1β, and TNF-α inflammatory proteins, as well as p-AMPK/AMPK, Beclin1, and LC3 II/LC3 I autophagy-related proteins in an AD mouse model. Additionally, the study found an increase in the expression levels of p-mTOR/mTOR and P62. Molecular docking analysis revealed positive binding results between ginsenosides and NLRP1. Lv J et al. [233] conducted a study to investigate the effects of ginsenoside treatment on scopolamine-induced memory deficits in mice. The study aimed to elucidate the potential mechanism of ginsenosides in regulating cholinergic transmission, inhibiting oxidative stress, and activating the ERK-CREB-BDNF signaling pathway.

#### 5.3.2. Notoginsenosides

The rhizome and fleshy root of Panax notoginseng are precious medicinal materials known for their hemostatic, blood-activating, anti-inflammatory, analgesic, and nourishing effects. Notoginsenoside, derived from the rhizome of Panax notoginseng, is a natural compound that can inhibit cell apoptosis and has neuroprotective effects. Initial studies have indicated that notoginsenosides can ameliorate AD by impeding tau protein phosphorylation and elevating BDNF expression levels [234]. Subsequently, Zhou N et al. [235] investigated the potential protective effects of Panax notoginseng Total Saponins (PNTS) against oxidative stress and cellular demise in an in vitro setting. The findings demonstrated that PNTS mitigated H_2_O_2_-induced cell death in primary rat cortical astrocytes. The protective impact of PNTS on astrocytes is associated with the activation of Nrf2, which enhances the downstream antioxidant system to diminish ROS. Furthermore, research conducted by Ma B et al. [236] demonstrated that notoginsenosides can enhance cell viability, decrease cell apoptosis, restore mitochondrial membrane potential, and impede the MAPK signaling pathway, thus mitigating the detrimental effects of Aβ. Recent investigations have revealed that notoginsenosides can diminish APP-Thr668 phosphorylation and BACE1 expression and enhance ADAM10 and insulin-degrading enzyme expression, ultimately decreasing Aβ production and augmenting Aβ degradation, thereby effectively safeguarding neurons and fostering cognitive abilities [237].

#### 5.3.3. Dioscin

Dioscin, derived primarily from medicinal plants of the Dioscoreaceae family (such as Japanese yam, dragon yam, and Chinese yam), are natural steroidal saponins that hold significant value in medicinal research. In recent years, there has been a gradual increase in research focused on these compounds. Zhang XS et al. [238] studied its neuroprotective effect on subarachnoid hemorrhage in mice and found that dioscin can inhibit inflammation, oxidative damage, and neuronal degeneration, as well as improve the cell viability of neurons and astrocytes. In addition, Guan L et al. [239] conducted a study on the therapeutic mechanism of dioscin in treating AD and discovered that these saponins exhibit promising anti-AD effects by regulating RAGE/NOX4-mediated oxidative stress and inflammation. This regulation leads to a significant improvement in the spatial learning and memory abilities of AD mice. The antioxidant mechanism involves the upregulation of Nrf2 and HO-1 expression levels, which in turn increases SOD and reduces MDA levels. Additionally, it downregulates the levels of inflammatory factors associated with the NF-κB inflammation pathway. Furthermore, dioscin also possess the ability to inhibit AChE. Collectively, these mechanisms reverse the histopathological changes observed in brain tissue. Zhang Z et al. [240] found that dioscin can effectively inhibit Aβ1-42 oligomer-induced neurotoxicity, reduce cell apoptosis and ROS production, and enhance the expression of the neuroprotective protein SIRT3. Moreover, dioscin induces the formation of autophagosomes and autolysosomes in HT22 cells, increases the levels of Beclin-1 and LC3 II, and decreases p62, thereby promoting the activation of the autophagy system.

**Table 3:** Summary of recent studies on the role and mechanisms of saponins natural plant molecules on AD BBB-related targets.

Natural Plant Molecules	Experimental Subjects	Possible Mechanisms	Target of Action	Reference
Ginsenosides	AD tree shrews	Regulating Wnt/GSK-3β/β-catenin signaling pathway	Astrocytes and microglia	Yang 2022 [228]
	D-gal induced rats	Regulating Nrf2-ARE pathway	Endothelial cells and microglia	Wang 2020 [229]
	Aβ-treated primary cultured rat hippocampal neurons	↓ CDK5 induced PPARγ phosphorylation	Microglia and astrocytes	Quan 2020 [231]
	APP/PS1 mouse	↑ NLRP1 inflammasome ↑ Autophagy function	Microglia and astrocytes	Li 2023 [232]
	Scopolamine-induced mice	Regulating cholinergic transmission ↓ Oxidative ↑ ERK-CREB-BDNF signaling pathway	Endothelial cells, microglia and astrocytes	Lv 2021 [233]
	APP/PS1 mouse	Regulating AMPK/Nrf2 signaling pathway	Endothelial cells and microglia	She 2023 [230]
Notoginsenosides	Okada acid-treated AD brain slices	↓ Tau protein phosphorylation ↑ BDNF	Endothelial cells and astrocytes	Wang 2013 [234]
	Aβ-induced PC12 neuronal cells	↓ MAPK signaling pathway	Astrocytes and microglia	Ma 2014 [236]
	Aβ-induced AD rats	↓ BACE1 ↑ ADAM10, IDE	Endothelial cells and microglia	Liu 2019 [237]
	H_2_O_2_-treated primary rat cortical astrocytes	Regulating Nrf2 signaling pathway	Endothelial cells and microglia	Zhou 2014 [235]
Dioscin	H_2_O_2_-treated SH-SY5Y cells and D-gal-induced C57BL/6J mice	Regulating the RAGE/NOX4 pathway	Endothelial cells and microglia	Guan 2022 [239]
	Aβ1-42-treated HT22 cells	Regulating SIRT3 and autophagy	Astrocytes and microglia	Zhang 2020 [240]

↓: downregulation or inhibition; ↑: upregulation or activation.

### 5.4. Others (Table 4)

#### 5.4.1. Astragaloside IV

Astragaloside IV (AS-IV) is the most potent bioactive component of Astragalus membranaceus, commonly referred to as the “super polysaccharide of Astragalus”. It can alleviate endothelial dysfunction, promote neurogenesis, angiogenesis, and neural function recovery, and increase the protein expression of BDNF and vascular endothelial growth factor (VEGF) [241]. Furthermore, it can prevent neurodegeneration by reducing mitochondrial damage and inhibiting astrocyte aging [242]. In vitro studies by He et al. [243] have shown that AS-IV inhibits the phosphorylation of NF-κB and p65, resulting in a reduction in the mRNA expression of IL-1β, COX-2, iNOS, and TNF-α in LPS-stimulated BV-2 cells. In mice, AS-IV has been found to decrease the formation and accumulation of Aβ plaques and improve cognitive impairment in AD mice. Another study by Chen et al. [244] suggests that AS-IV may inhibit microglial activation and downregulate NADPH expression, thereby improving neuroinflammation and neuronal damage in Aβ-induced mice. Furthermore, AS-IV, as a potential PPARγ agonist, has been shown to effectively inhibit the activity of BACE1 and reduce Aβ levels, making it a promising alternative to the PPARγ antagonist GW9662 [245]. Experimental data from Chang et al. [246] indicate that AS-IV may positively impact AD treatment by regulating the PI3K/AKT and MAPK (or ERK) pathways. This regulation is supported by the inhibition of caspase-3 expression, tau hyperphosphorylation, loss of dendritic marker MAP2, and regulation of mitochondrial membrane potential, all of which are consistent with the effects of corresponding pathway inhibitors.

#### 5.4.2. Schisandrin

Schisandra chinensis, a Magnoliaceae plant primarily distributed in China, has been extensively utilized in Asia. In recent years, numerous scholars have been studying the active components of Schisandra chinensis. Among them, schisandrins are natural compounds extracted from the fruit of Schisandra chinensis. Previous studies have indicated that schisandrins possess antioxidative, anti-inflammatory, anti-fatigue, neurotransmitter regulation, anti-aging, and cardiovascular and cerebrovascular protection effects. Recent studies have shown that it can influence the progression of AD by modulating peripheral plasma lipid metabolism through the brain [247]. In a study by Li Q et al. [248], it was discovered that schisandrin partially reversed neuronal apoptosis induced by Aβ, which is closely associated with the inhibition of NLRP1 inflammasome activation. Furthermore, in a study conducted by Giridharan VV et al. [249], the effects of schisandrin on Aβ-induced rats were examined, revealing its regulatory functions in various aspects including oxidative nitrosative stress, the activation of glial cells, RAGE/NF-κB/MAPK activation, and autophagy.

#### 5.4.3. Salvianic Acid A

Salvianic acid A (SalA) is one of the major water-soluble pharmacological com-ponents of Salvia miltiorrhiza. It belongs to the class of phenolic aromatic acids and exhibits a wide range of pharmacological effects in cardiovascular and cerebrovascular diseases, with the most significant being its antioxidative activity and improvement of microcirculation. In a study by Zhao et al. [250], the therapeutic impact of SalA in neurodegenerative diseases was summarized. This impact includes the inhibition of Aβ aggregation and fibril formation, reduction of Aβ neurotoxicity, decrease in tau protein hyperphosphorylation, prevention of neuroinflammation and oxidative damage, inhibition of cell apoptosis, restoration of mitochondrial dysfunction, and promotion of neuronal regeneration. Another study by Lee et al. [251] discovered that SalA can diminish the activation of microglia and astrocytes in AD mice. It also significantly reduces the expression levels of iNOS and COX-2 as well as the levels of thiobarbituric acid reactive substances. Furthermore, SalA notably inhibits the decline in choline acetyltransferase and BDNF protein levels. In terms of reducing Aβ, SalA achieves this by inhibiting BACE1 expression and regulating APP processing. Additionally, it can also inhibit GSK3β signaling and oxidative stress, while increasing the levels of ADAM10 and soluble amyloid precursor proteins alpha (sAPPα) in cells [252,253].

**Table 4:** Summary of recent studies on the role and mechanisms of other natural plant molecules on AD BBB-related targets.

Natural Plant Molecules	Experimental Subjects	Possible Mechanisms	Target of Action	Reference
Astragaloside IV	5xFAD mouse and LPS-stimulated BV-2 cells	↓ NF-κB signaling pathway	Astrocytes and microglia	He 2023 [243]
	Aβ-induced AD mice	↓ Microglial activation ↓NADPH oxidase protein expression	Endothelial cells and microglia	Chen 2021 [244]
	APP/PS1 mouse	↑ PPARγ pathway ↓ BACE1	Endothelial cells and Astrocytes	Wang 2017 [245]
	Aβ25-35-induced AD rat	Regulating PI3K/AKT and MAPK (or ERK) pathways	Microglia and astrocytes	Chang 2016 [246]
Schisandrin	Aβ25-35–APP/PS1 mouse	↓ NLRP1	Microglia and astrocytes	Li 2021 [248]
	Aβ-induced neuronal cells in rat brain	↓ RAGE/NF-κB/MAPK ↑ HSP/Beclin	Endothelial cells, microglia, and astrocytes	Giridharan 2015 [249]
Salvianic acid A	Aβ25-35-induced AD mice	↓ Inflammation ↓ Oxidative stress	Endothelial cells and Astrocytes	Lee 2013 [251]
	SH-SY5Y-APPsw cell	↓ Oxidative stress ↓ GSK3β signal transduction	Endothelial cells and Astrocytes	Tang 2016 [252]
	N2a-mouse and H4-human neuroglioma cell lines expressing SwedAPP	↓ BACE1	Endothelial cells and microglia	Durairajan 2017 [253]

↓: downregulation or inhibition; ↑: upregulation or activation.

## 6. Discussion and Conclusions

Natural plant molecules have a wide range of sources, and the medicinal components mentioned in the above content are widely present in vegetables and fruits consumed in daily diets. However, they are most abundant in many plants and herbal medicines, and their extraction processes are more advanced. In particular, traditional Chinese herbal medicines, which are currently the focus of intense research, have great potential in terms of their active ingredients in modern studies. Thousands of years ago, the therapeutic effects of these natural products were already recognized through human medical experiences, which involved the use of animals and plants from nature for drug therapies and dietary treatments. Returning to the origins, our focus is now directed towards these animal and plant medicinal materials, particularly herbal plants. These materials are not only widely available but also rich in medicinal components. Some of them are even referred to as natural antioxidants, which serve as important sources for new drug development and as effective means for preventing and treating diseases. Recently, an increasing number of natural plant molecules have been discovered to have a significant impact on AD, with the key mechanism being closely associated with the regulation of the BBB.

Previous studies have clearly demonstrated a close relationship between the pathogenesis of AD and the BBB. Several studies indicate that age-related changes in the BBB may precede the onset of AD in the elderly population [254]. If this is the case, further emphasis can be placed on the prevention of AD in the elderly population. Despite the ample experimental support and extensive research on the underlying mechanisms, there is a scarcity of reports on drugs that directly target the BBB. The poor solubility and low bioavailability of these drugs make it challenging to penetrate the BBB. Consequently, in recent years, immunotherapy, gut microbiota intervention, and physical exercise have garnered attention [255]. However, the benefits they offer require a longer duration and patience. Therefore, Hao Z et al. [256] proposed alleviating AD through exercise targeting the brain–gut axis, as well as the prevention and treatment of AD through exercise combined with polyphenols. Currently, numerous studies have been conducted in the field of nanotechnology to achieve targeted drug delivery and enhance drug bioavailability, resulting in significant advancements in nanomedicine and intranasal administration [257,258]. However, breakthrough results are still lacking, and the focus of nanotechnology applications is gradually shifting towards natural molecules [259]. Despite the lack of separate reports on the regulatory effects of the aforementioned plant molecules on the BBB, our research on their treatment of AD has revealed their ability to regulate BBB-related pathways, suggesting their potential for AD treatment and prevention. Thus, investigating the regulation of BBB-related targets using natural plant molecules could offer a novel approach to intervene in AD progression and enhance cognitive function.

In conclusion, the regulatory mechanism of natural plant molecules on the BBB in AD is both extensive and complex. Nevertheless, there is no doubt that natural plant molecules possess strong targeting capabilities as drugs for AD treatment. They can effectively penetrate the BBB and offer inherent advantages in maintaining its integrity and regulating its function. They promote the transport of Aβ, reduce neurotoxicity, protect neurons from damage, and improve the pathological features of AD through anti-inflammatory, antioxidant, signal pathway regulation, and inhibition of cell apoptosis. These effects have been demonstrated in animal and cell experiments including the restoration of learning and memory abilities. However, there is currently no clear research on the toxic side effects associated with these plant molecules. Although these molecules are relatively reliable, it is still necessary to conduct toxicological experiments for detection. Subsequent clinical trials are needed to further confirm the multi-pathway targeting effect of natural plant molecules on the BBB in AD patients. Additionally, the combined effect of multiple molecules should be observed based on the research of individual plant molecules. Furthermore, general pharmacology, pharmacokinetics, and pharmacogenetics should study issues such as dosage form and metabolism time. This is a complex and challenging task that faces various problems, including trial design, trial period definition, ethical review, and technical funding support. Therefore, more effort is required to maximize the biological activity of these natural plant molecules on the AD BBB and develop more effective therapeutic drugs.

## Data Availability

All data are available in the manuscript and they are shown in the figures and tables.

## Abbreviations

AD	Alzheimer’s disease
Aβ	Amyloid β-protein
NFT	Neurofibrillary tangle
BBB	Blood–brain barrier
NVU	Neurovascular unit
CNS	Central nervous system
BMECs	Brain microvascular endothelial cells
TJ	Tight junction
BDNF	Brain-derived neurotrophic factor
AQP4	Aquaporin-4
PDGF-BB	Platelet-derived growth factor-BB
BM	Basement membrane
ECM	Extracellular matrix
MAPK	Mitogen-activated protein kinase
BACE1	β-amyloid precursor protein cleaving enzyme 1
LRP-1	Low-density lipoprotein-receptor-related protein 1
RAGE	Receptor for advanced glycation endproducts
CAA	Cerebral amyloid angiopathy
ZO-1	Zonula occludens protein 1
PECAM-1/CD31	Platelet endothelial cell adhesion molecule-1
ICAM	Intercellular adhesion molecule
MMPs	Matrix metalloproteinases
NADPH	Nicotinamide adenine dinucleotide phosphate
ETS	Erythroid transformation-specific
NF-κB	Nuclear factor kappaB
JNK	c-Jun N-terminal kinase
PI3K	Phosphoinositide 3-kinase
Akt	Protein kinase B
NMDA	N-methyl-D-aspartic acid
AMPA	Alpha-amino-3-hydroxy-5-methyl-4-isoxazole propionic acid
ROS	Reactive oxygen species
ERK	Extracellular signal-regulated kinase
NOX	NADPH oxidase
NLRP3	N=Nucleotide-binding domain-like receptor protein 3
PPARγ	Peroxisome proliferator-activated receptor gamma
COX-2	Cyclooxygenase-2
iNOS	Inducible nitric oxide synthase
GSK-3β	Glycogen synthase kinase-3β
PTS	Pterostilbene
Bcl-2	B-cell lymphoma-2
Bax	BCL-2-associated X
MDA	Malondialdehyde
SOD	Superoxide dismutase
GSH	Glutathione
cAMP	Cyclic adenosine monophosphate
CREB	cAMP response element-binding protein
PSD95	Postsynaptic density protein 95
PDE4	Phosphodiesterase4
Nrf2	Nuclear factor erythroid 2-related factor 2
AMPK	Adenosine 5′-monophosphate (AMP)-activated protein kinase
Ht22	Mouse hippocampal neuronal cells
GA	Gallic acid
ADAM10	A disintegrin and metalloprotease 10
LTP	Long-term potentiation
EGCG	Epigallocatechin gallate
LPS	Lipopolysaccharide
TLR4	Toll-like receptor 4
mTOR	Mammalian target of rapamycin
LC3	II microtubule-associated proteins light chain 3 II
PKA	Protein kinase A
TrkB	Tropomyosin-related kinase B
AChE	Acetylcholinesterase
Keap1	Kelch-1ike ECH-associated protein l
GSSG	Glutathione disulfide
BAI	Baicalin
Cyt-C	Cytochrome-c
Sal	Salidroside
D-gal	D-galactosamine
SIRT1	Sirtuin 1
NeuN	Neuron-specific nuclear protein
ER	Endoplasmic reticulum
ICA	Icariin
Wnt	Wingless-related integration site
PFC	Prefrontal cortex
MAP2	Microtubule-associated protein 2
PNTS	Panax notoginseng Total Saponins
HO-1	Heme oxygenase 1
AS-IV	Astragaloside IV
SalA	Salvianic acid A
